# Fever in a patient with mild acute pancreatitis caused by ulinastatin injection: A case report

**DOI:** 10.1097/MD.0000000000046808

**Published:** 2026-01-02

**Authors:** Tong Wu, Linzhen Li, Yan Zhang

**Affiliations:** a Department of Gastroenterology, The First Affiliated Hospital of Wannan Medical College, Wuhu, Anhui Province, China.

**Keywords:** acute pancreatitis, fever, ulinastatin injection

## Abstract

**Rationale:**

The case describes a patient with mild acute pancreatitis (AP) who developed a persistent high fever during ulinastatin treatment. This highlights the possibility of drug-induced fever, which is a rare but important adverse reaction of ulinastatin and is often overlooked in clinical practice.

**Patient concerns:**

A 58-year-old male patient with mild AP had a persistent high fever during the treatment with ulinastatin, but there was no evidence of infection.

**Diagnoses:**

Mild AP, ulinastatin-related drug fever.

**Interventions:**

The initial conservative treatment for AP included fasting, rehydration and taking ulinastatin. Antibiotics (ceftriaxone, later changed to imipenem) were used for suspected infection. After excluding infection, ulinastatin was discontinued.

**Outcomes:**

Within 48 hours of stopping ulinastatin, the patient’s fever symptoms were relieved and his subsequent condition stabilized.

**Lessons:**

This case highlights the importance of considering drug-induced fever in clinical practice. Recognizing it promptly and discontinuing the drug can prevent unnecessary interventions and improve outcomes.

## 1. Introduction

Acute pancreatitis (AP) is a prevalent acute abdominal disease with a complex pathogenesis in which the inflammatory response plays a key role. Ulinastatin, as a serine protease inhibitor, can alleviate inflammation and tissue damage, and reduce the mortality of patients.^[1]^ However, ulinastatin may also cause some adverse reactions during clinical application. This article reports a case of fever during the treatment of mild AP with ulinastatin, aiming to improve clinicians’ understanding of the adverse reactions of the drug and provide a reference for the safe and rational use of the drug.

## 2. Case report

**Figure 1.**
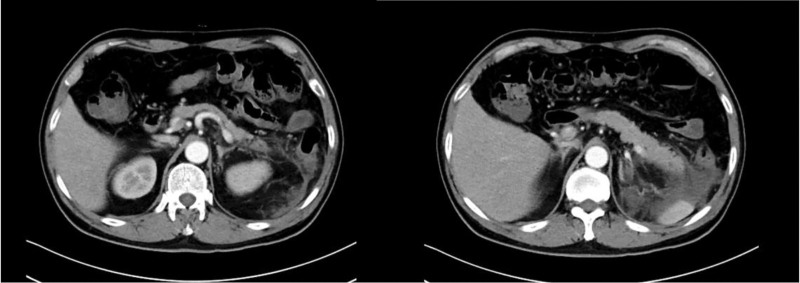
Whole abdomen enhanced CT showed exudation and effusion of pancreatic body and tail. CT = computed tomography.

A 58-year-old male was admitted to the hospital on March 15, 2025 for left upper abdominal pain after drinking for 2 days. The patient was in good health normally, with no history of chronic diseases such as hypertension, diabetes, coronary heart disease. He had no history of drug allergies such as penicillins, cephalosporins, sulfonamides, and no history of food allergies such as seafood, mango. There was no history of drug allergies or autoimmune diseases such as rheumatoid arthritis or systemic lupus erythematosus in the family. The patient was accompanied by nausea, fatigue and anorexia. Vital signs were normal. On physical examination, he appeared sick, with moderate left epigastric tenderness. A full abdominal computed tomography (CT) scan and blood amylase examination in another hospital indicated AP. A diagnosis of AP was made, and the patient was treated conservatively, including fasting, rehydration, inhibit digestive fluid secretion, and acid suppression. Ulinastatin (100,000 IU/time, intravenous drip, 1 time/24 hours) was also used. On the day of admission, the patient had fever with a temperature of 38.4℃, therefore, ceftriaxone was given anti-infective treatment. After 4 days of treatment, the body temperature did not decrease significantly. Routine blood tests showed that the white blood cells were 13.9 × 10^9^/L, the percentage of neutrophils was 80.6%, and the procalcitonin was normal. Ceftriaxone was changed to imipenem. However, after 3 days of imipenem, the patient’s body temperature did not drop, but rose to 39.5℃. Chest CT did not reveal any obvious inflammatory lesions, and no bacteria or fungi were cultured from the blood. The procalcitonin was normal after repeated tests, and the respiratory tract pathogen examination only showed that the previous immunoglobulin G antibody had been positive. Whole abdomen enhanced CT showed exudation and effusion of pancreatic body and tail, no signs of pancreatic necrosis and infection (Fig. 1). Therefore, we suspected that the fever was not caused by an infection, but rather a drug-induced fever, and the suspected drug ulinastatin was discontinued on March 21, 2025. On March 22, the maximum body temperature of the patient dropped to 37.4℃, and the body temperature was normal on March 23. Imipenem was discontinued on March 24, and was discharged on March 28 with no recurrence of fever.

## 3. Discussion

As a common clinical symptom, fever has a complex and diverse etiology, mainly including infectious diseases, autoimmune rheumatic diseases, malignant tumors and postoperative reactions. For patients with persistent fever, clinicians need to carry out comprehensive infection screening and investigation of various potential etiologies. However, when the routine examination results are all negative and the fever symptoms persist, clinical workers should consider the possibility of drug-induced fever. Drug fever is a particular concern in the field of adverse drug reactions. This condition is frequently misdiagnosed and often goes unnoticed. An epidemiological study in Korea, grounded in national health insurance claims data, has revealed that an overall incidence of drug fever was about 91/10 million.^[2]^ A multitude of pharmaceutical agents have been demonstrated to be associated with drug fever, mainly including antibiotics, antineoplastics, antiepileptics, psychotropic drugs, and hypoglycemic drugs.^[3]^ As the adverse drug reaction monitoring system undergoes continuous improvement, the list of drugs associated with drug fever continues to expand. It requires that clinicians should be vigilant in the diagnosis and treatment process, and drug fever should be included in the routine differential diagnosis of fever of unknown origin.

It should be noted that ulinastatin, as a protease inhibitor, has shown significant clinical value in the treatment of AP, but it also has the potential risk of causing drug fever. A number of clinical studies have demonstrated the efficacy and safety of ulinastatin in reducing the rate of complications, shortening hospitalization time and improving inflammatory indices.^[1,4,5]^ However, this case report shows that the patient developed fever after using ulinastatin, without clinical symptoms of infection such as cough, expectoration, frequent urination. Multiple blood culture results were negative, and no pathogenic bacteria growth was observed. No white blood cells were found in the urine routine test, and nitrite was negative. Respiratory pathogen examination showed that the positive immunoglobulin G antibody of mycoplasma pneumoniae indicated previous infection, while antibodies related to acute infections such as influenza virus and respiratory syncytial virus were all negative. The serum levels of 1,3-β-D-glucan (*G* test, <70 pg/mL) and galactomannan (GM test, <0.5) were both within the normal reference intervals, providing no evidence for fungal infection. Chest CT demonstrated no definite signs of pulmonary infection. Abdominal ultrasound revealed no evidence of biliary tract infection, and whole abdomen enhanced CT showed no signs of pancreatic necrosis or abscess formation. These findings systematically ruled out the possibility of infectious fever. Furthermore, his temperature returned to normal within 48 hours after stopping the drug, which is consistent with the typical clinical features of drug fever. Although the drug instructions of ulinastatin have clearly listed its adverse reactions such as chills, fever and other systemic damage, the specific mechanism of fever has not been fully clarified. Generally speaking, ulinastatin is well-tolerated, with a low frequency of adverse events.^[6]^ Studies have shown that the most common adverse reactions to intravenous ulinastatin include dizziness, pain at the injection site, and leukopenia.^[7,8]^ As far as we know, there are currently no case reports of ulinastatin-related drug fever at home and abroad. This case is reported for the first time, filling the gap of this adverse reaction in clinical literature. This characteristic – being documented in the package insert yet supported by scarce clinical documentation – maked febrile reactions an easily overlooked potential risk. Given that ulinastatin is a protein-based therapeutic, it has the potential to induce immune-mediated reactions. The observed febrile response is hypothesized to be associated with drug allergy or anaphylactoid reactions. However, direct evidence supporting this association remains lacking. This suggests that clinicians need to enhance their awareness and surveillance of such adverse reactions. In clinical practice, doctors should ask the patient’s history of drug and food allergies in detail. Medications should be selected carefully to ensure the safety and efficacy of treatment. Closely observe the patient’s response during the process of medication administration, and regularly perform laboratory tests such as blood routine, liver and kidney function, in order to promptly detect adverse drug reactions and take appropriate measures.

During a post-discharge follow-up, the patient expressed: “The fever during the later stage of my hospitalization caused me significant anxiety. Despite repeated examinations, the doctors could not identify an infection. The fever subsided only after ulinastatin was discontinued. It had not occurred to me that drugs could also cause fever. I hope that in the future, doctors can inform patients in advance about potential adverse reactions to drugs. I will also pay closer attention to changes in my body and cooperate more actively with the treatment.” This feedback underscores the importance of enhancing patient education prior to medication administration. Healthcare providers should proactively inform patients about potential adverse drug reactions to help establish awareness in advance. This practice can effectively alleviate patient anxiety when unexplained symptoms arise, thereby strengthening trust in treatment and improving adherence.

Our study has some limitations. Firstly, as this report is a single-case observation, it is unable to quantify the incidence of ulinastatin-induced drug fever. Establishing a definitive causal relationship between ulinastatin and drug fever requires future validation through multicenter, large-sample adverse drug reaction monitoring data. Secondly, as readministration of ulinastatin could have induced a recurrence of high fever in the patient, a drug rechallenge was precluded on ethical grounds. Given that a positive rechallenge is considered the gold standard for confirming drug-induced fever, this absence may affect the robustness of our diagnosis.

In summary, ulinastatin is a significant drug for the treatment of AP. The emergence of adverse reactions such as drug fever suggests that we need to maintain a prudent attitude in clinical application. It is recommended that clinicians should take into account the patient’s condition and individual differences when formulating treatment plans. Implementing individualized medication strategies will improve the therapeutic efficacy and reduce the occurrence of adverse reactions, ensuring the safety of patients’ medication.

## Acknowledgments

The authors would like to thank the patient for granting permission to publish this case report and are grateful to the clinical team for their support.

## Author contributions

**Writing – original draft:** Tong Wu.

**Writing – review & editing:** Yan Zhang, Linzhen Li.
